# Genome sequences of two *Klebsiella* phages isolated from wastewater treatment samples that infect a clinical *Klebsiella* isolate

**DOI:** 10.1128/MRA.00836-23

**Published:** 2024-12-17

**Authors:** Jessica M. Lewis, Daniel K. Arens, Abraham Quaye, Diana G. Calvopina-Chavez, Kyson T. Jensen, Ashley K. Miller, Melinda M. Moss, M. Elizabeth Warren, Justina P. Tavana, Steven M. Johnson, Julianne H. Grose

**Affiliations:** 1 Microbiology and Molecular Biology Department, Brigham Young University, Provo, Utah, USA; Portland State University, Portland, Oregon, USA

**Keywords:** *Klebsiella oxytoca*, bacteriophage, ValerieMcCarty03, ValerieMcCarty04, phage therapy, T4-like, jiaodavirus

## Abstract

We announce the complete genomes of *Klebsiella* phages ValerieMcCarty03 and ValerieMcCarty04 isolated from wastewater treatment plant samples that infect a multidrug-resistant *Klebsiella oxytoca* clinical isolate. These phages belong to the T4-like cluster and fall within the *Jiaodavirus* genus.

## ANNOUNCEMENT

The fight against antibiotic-resistant infections is one of the greatest global health challenges to date and mandates the discovery of alternative treatments (1). One therapy being explored is the use of bacteriophages which are the natural predators of bacteria. *Klebsiella pneumoniae* causes a third of all Gram-negative infections (2) and *Klebsiella oxytoca* is establishing a presence among highly susceptible immunocompromised patients (3, 4). Herein, we describe the full genomic sequences of two *Klebsiella* phages that infect a *Klebsiella* clinical isolate derived from a diabetic ulcer.

ValerieMcCarty03 and ValerieMcCarty04 were isolated from raw sewage sampled from Utah Valley, Utah, USA (Table 1) and propagated in the presence of a *Klebsiella* sp., clinical isolate, which was verified as a likely *K. oxytoxa* strain by 16S sequencing in the Grose lab (the top BLASTN hit was K. *oxytoxa* strain PWX3 with 99.89% identity over the 939 bp (5)). Briefly, candidate phages were grown via enrichment by adding wastewater treatment plant influx samples (0.5 mL), lysogeny broth (LB, 4 mL), and overnight culture (0.5 mL) of the clinical *Klebsiella* isolate and allowed to propagate at 37°C for 48 h. Amplified phages were then harvested by centrifugation (6,000 × *g* for 20 min) and 50 µL of the lysate was plated with 0.5 mL of fresh bacteria in 5 mL of LB top agar. One plaque per sample was picked and purified by plating three subsequent times on the clinical isolate. High titer lysates (>10^8^ pfu/mL) were generated by adding the purified phage plaque to 1 mL bacteria and 5 mL of LB at 37°C for 24–48 h and harvesting using centrifugation.

**TABLE 1:** Data specifications of ValerieMcCarty03 and 04

Phage name	GenBank accession no.	SRA accession no.	Total no. of reads	Min–Max (average) fold coverage	Length (bp)	GC content (%)	Sewage sample GPS
ValerieMcCarty03	OR106137	SRR25248969	413240	183–298 (250)	168,196	40	40°10′N 111°40′W
ValerieMcCarty04	OR271596	SRR25265492	358111	98–348 (226.4)	166,087	39.8	40°10′N 111°40′W

Phage DNA extraction from phage lysate and subsequent sequencing library prep was performed using the Norgen Phage DNA isolation kit (Norgen Biotek, Canada) and the Illumina TruSeq DNA Nano kit, respectively. DNA sequencing was carried out on the Illumina iSeq 100 platform with 150 bp paired-end reads. The genomes (see Table 1) were assembled using *de novo* assembly with Geneious (BioMatters) version 2022.2.1 for ValerieMcCarty03 and 8.0.5 for ValerieMcCarty04 at default settings without trimming (6). Both circularized upon assembly. GeneMarkS version 4.28 (7) was used to predict the coding potential of the finalized genomes which were then annotated using DNA Master version 5.23.6 (8). Predicted gene starts from DNA Master were extended when necessary to fully cover the coding potential and the default settings were used for all programs.

Whole-genome dotplot (9) comparison with top BlastN (5) hits exceeded 50% sequence similarity (>72%) with T4-like phages which qualifies them to be classified within the T4 cluster by our previously published classification system for phages that infect *Enterobacterales* (10). Because these are T4-like phages and T4 is known to package DNA by the headful mechanism, the start of each phage genome was modeled after the well characterized T4 genome (AF158101). Further classification of these phages as *Jiaodaviruse*s is supported by their closest relatives from BlastN search of the nucleotide collection (nr/nt), with ValerieMcCarty03 sharing 96.35% identity (97% coverage) with phiKp_22 (LC768492) and ValerieMcCarty04 sharing 99.59% identity (99% coverage) with vB_Kte_K65PH164 (OY979421).

## Data Availability

Bioproject PRJNA993706 was used for both phages under BioSample accession nos. SAMN36408255 (ValerieMcCarty03) and SAMN36417985 (ValerieMcCarty04). GenBank and SRA accession numbers are provided in Table 1.
